# Oxyradical Stress, Endocannabinoids, and Atherosclerosis

**DOI:** 10.3390/toxics3040481

**Published:** 2015-12-03

**Authors:** Anberitha T. Matthews, Matthew K. Ross

**Affiliations:** Center for Environmental Health Sciences, Department of Basic Sciences, College of Veterinary Medicine, Mississippi State University, P.O. Box 6100, Mississippi State, MS 39762, USA; E-Mail: anberitha@gmail.com

**Keywords:** reactive oxygen species, NADPH oxidase, 2-arachidonoylglycerol, anandamide, cardiovascular disease, atherogenesis

## Abstract

Atherosclerosis is responsible for most cardiovascular disease (CVD) and is caused by several factors including hypertension, hypercholesterolemia, and chronic inflammation. Oxidants and electrophiles have roles in the pathophysiology of atherosclerosis and the concentrations of these reactive molecules are an important factor in disease initiation and progression. Overactive NADPH oxidase (Nox) produces excess superoxide resulting in oxidized macromolecules, which is an important factor in atherogenesis. Although superoxide and reactive oxygen species (ROS) have obvious toxic properties, they also have fundamental roles in signaling pathways that enable cells to adapt to stress. In addition to inflammation and ROS, the endocannabinoid system (eCB) is also important in atherogenesis. Linkages have been postulated between the eCB system, Nox, oxidative stress, and atherosclerosis. For instance, CB_2_ receptor-evoked signaling has been shown to upregulate anti-inflammatory and anti-oxidative pathways, whereas CB_1_ signaling appears to induce opposite effects. The second messenger lipid molecule diacylglycerol is implicated in the regulation of Nox activity and diacylglycerol lipase β (DAGLβ) is a key biosynthetic enzyme in the biosynthesis eCB ligand 2-arachidonylglycerol (2-AG). Furthermore, Nrf2 is a vital transcription factor that protects against the cytotoxic effects of both oxidant and electrophile stress. This review will highlight the role of reactive oxygen species (ROS) in intracellular signaling and the impact of deregulated ROS-mediated signaling in atherogenesis. In addition, there is also emerging knowledge that the eCB system has an important role in atherogenesis. We will attempt to integrate oxidative stress and the eCB system into a conceptual framework that provides insights into this pathology.

## 1. Introduction

Atherosclerosis is the major underlying pathology that causes cardiovascular disease (CVD). Despite considerable investment in the development of new therapies, atherosclerosis remains the leading cause of morbidity and mortality in industrialized societies. At its heart, atherosclerosis is a chronic inflammatory disease caused by inflammatory cascades, dysregulated lipid metabolism, and oxidative stress [1]. Exogenous and endogenous small molecules—and the enzymes that produce or degrade these molecules—have important roles in these processes. This review will focus on reactive oxygen species (ROS), their important roles in intracellular signaling pathways, and the impact that dysregulated ROS signaling has in pathophysiological processes, such as atherogenesis. An operational definition for ROS is that they are redox active chemicals, which include oxygen-containing compounds with an unpaired electron, such as superoxide (O_2_^·−^), and nonradical oxygen-containing compounds, such as hydrogen peroxide (H_2_O_2_). In addition, reactive nitrogen species, such as nitric oxide, can also be broadly included under the umbrella of ROS. Although atherosclerosis is a multifactorial disease involving endothelial cell dysfunction, hyperlipidemia, inflammation, hypertension, and imbalances in ROS formation and degradation, there is also emerging knowledge that the endocannabinoid (eCB) system has an important role in atherogenesis. Thus, the role of the eCBs will be discussed in the context of inflammation and atherosclerosis.

## 2. Oxyradical Stress, NADPH Oxidase, and Atherogenesis

Because of the unique chemistry of oxygen (O_2_) and its ability to harvest the rich energy reserves found in nutrients, such as glucose and lipids, life is able to exist on our planet. The ability of living organisms to use oxygen, however, has costs because ROS are significant by-products of aerobic respiration. In some contexts, ROS have beneficial properties that enable the innate immune system to confront microbial invaders. On the other hand, excess ROS can result in macromolecule damage and cell death. Therefore, excess ROS are important mediators of cellular and tissue injury, which in part contributes to disease development. Oxidative stress has been implicated as a causative factor in several diseases and aging [2,3,4]. Because of the development of chemical tools that are used as selective probes to detect and quantify specific ROS, such as O_2_^·−^ and H_2_O_2_, the biochemical pathways that shape the landscape of oxidative stress in living cells are becoming clearer [5]. This has enabled causal inferences instead of mere associations to be made for specific oxyradical species in various physiological processes, which has provided important insights into vascular biology and disease.

**Figure 1:**
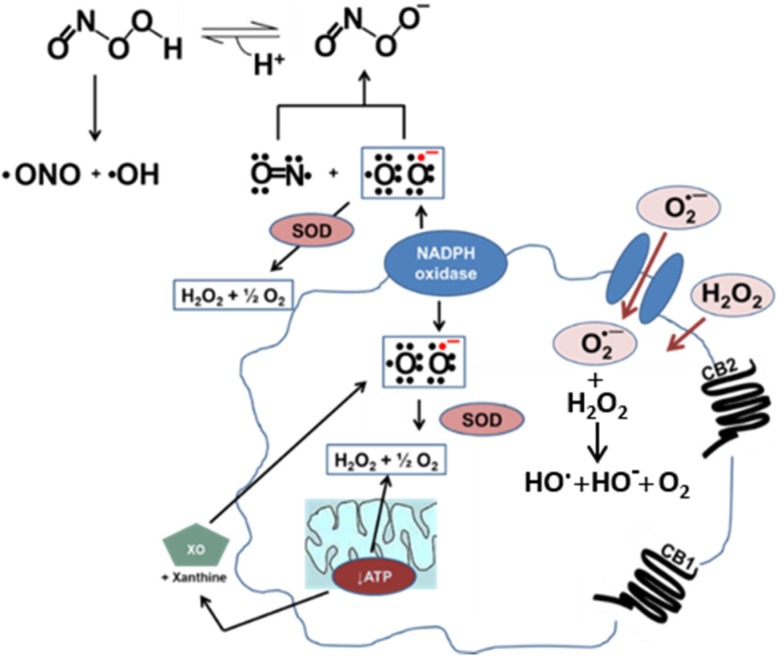
Oxidative stress in vascular cells. Nitric oxide (NO) and superoxide (O_2_^·−^) combine to make peroxynitrite (ONOO^−^) at a diffusion-limited rate. Superoxide is rapidly metabolized by superoxide dismutase (SOD) present in mitochondria, cytoplasm, and extracellular space. Alternatively, xanthine oxidase (XO) can produce superoxide as a byproduct of its activity. In a diseased vessel, these molecules can become toxic to the cell. Because of its negative charge O_2_^·−^ crosses lipid membranes via ion channels, whereas H_2_O_2_ can passively diffuse through the lipid bilayer. Activation of the CB_1_ receptor can enhance ROS and pro-inflammatory cytokine production. On the other hand, activation of the CB_2_ receptor is a protective mechanism due to the increased production of anti-inflammatory cytokines.

ROS-producing enzymes such as NADPH oxidase (Nox), xanthine oxidase, dysfunctional endothelial nitric oxide synthase, cytochrome P450 monooxygenase, and lipoxygenase are found in vascular cells, including endothelial cells, macrophages, and smooth muscle cells. These enzymes produce oxyradicals (O_2_^·−^) and oxy-nonradicals (H_2_O_2_) either as primary products or as by-products of their enzymatic activity. The incomplete reduction of oxygen during mitochondrial respiration also results in O_2_^·−^ production. In addition, myeloperoxidase catalyzes the reaction between H_2_O_2_ and chloride, which generates the powerful oxidant hypochlorite (HOCl). These reactions are described in Figure 1. Nox is the only oxidoreductase that produces O_2_^·−^ as its primary end product, thus it was given the moniker “professional ROS producer” [6]. Nox is typically inactive in circulating immune cells, such as neutrophils and monocytes, but in the presence of pro-inflammatory cytokines, such as IL-1β and TNF-α, it becomes activated. A multi-subunit complex is formed, which includes membrane embedded Nox2-p22^phox^ catalytic subunits and soluble regulatory subunits p47^phox^, p67^phox^, p40^phox^, and Rho GTPase (Rac1 or Rac2), resulting in the assembly of the Nox holoenzyme [6]. Active Nox catalyzes the one-electron reduction of molecular oxygen by NADPH, forming O_2_^·−^, which has a limited diffusion radius in the cell, because it is rapidly converted to H_2_O_2_ and oxygen (dismutation) either spontaneously or via superoxide dismutase (SOD). Furthermore, due to its negative charge, O_2_^·−^ cannot passively diffuse through lipid membranes, but it can pass through anion channels and aquaporins to access different compartments [7]. Although O_2_^·−^ *per se* is not reactive with cell macromolecules, SOD rapidly reduces its concentration in cells, which is important because this minimizes the Fe^3+^-catalyzed coupled reaction (Haber-Weiss) between O_2_^·−^ and H_2_O_2_ that produces hydroxyl radicals, hydroxide ion, and oxygen (Figure 1). First, O_2_^·−^ donates an electron to Fe^3+^ to generate Fe^2+^ and O_2_, then H_2_O_2_ reacts with Fe^2+^ to yield the hydroxyl radical and hydroxide ion (regenerating Fe^3+^, thus the net reaction is catalyzed by ferric ion). As compared with O_2_^·−^, the hydroxyl radical is highly reactive and abstracts hydrogen atoms from proteins, DNA, and lipids (mainly unsaturated fatty acids) in the vicinity of its production, thus generating more free radicals and propagating oxidative stress [1].

Whereas excess concentrations of O_2_^·−^ and ROS have obvious toxic properties, these species also have fundamental roles in signaling pathways that enable cells to adapt to stress [8,9]. There are several different isoforms of Nox expressed in cells and these enzymes have important roles in cellular homeostasis. For instance, macrophages express an abundant amount of Nox2. Nox2 is activated in response to a variety of physiological stimulants such as insulin, angiotensin II, and sheer stress. The O_2_^·−^ that is generated can act as a mild reductant because it surrenders an electron to an appropriate acceptor—for example, it can either reduce Fe^3+^ to Fe^2+^ or a second molecule of O_2_^·−^ to H_2_O_2_ [8,10]. Signaling pathways activated by O_2_^·−^-derived ROS include the stress kinase ERK1/2, which is involved in cell differentiation; JNK-MAPK, which is involved in the regulation of inflammation and cell death; NFκB, a transcription factor for inflammatory and anti-apoptotic genes; Akt, which is involved in regulating metabolic homeostasis; and Ras, Rac, and p38, which regulate several cellular functions such as proliferation, apoptosis, and inflammatory gene expression [9]. Nox2 is implicated in the development of atherogenesis and vascular remodeling [11,12,13]. For example, genetic deletion of Nox2 in the high-fat-diet-fed ApoE^−/−^ mouse model (ApoE^−/−^Nox2^−/−^) caused a reduction in atherosclerotic lesions compared with control ApoE^−/−^ mice [14]. This finding supports the notion that pharmacological inhibition of Nox might be an attractive strategy to reduce atherosclerosis [15]. In addition, vascular endothelial growth factor-induced angiogenesis and neovascularization was impaired in Nox2^−/−^ mice, which implicates Nox2 in wound healing and the generation of vessels [16].

A pathological consequence of the excess production of oxidants in the vascular space is the buildup of oxLDL and other oxidized biomolecules in the intima of the vessel wall [1,17]. LDLs entrapped in the intimal space are targets of oxyradical fluxes, leading to the chemical modification of lipids and apoproteins in the LDL particle. The resulting oxLDL particles are a hallmark lesion of atherosclerosis. Truncated oxidized phospholipids, such as phosphatidylcholine (oxPC_CD36_), are detected in atherosclerotic plaques and in the circulation of hyperlipidemic subjects [18,19]. Circulating monocytes then migrate into the intimal space of the vessel wall and in the presence of macrophage-colony stimulating factor (M-CSF) can differentiate into macrophages. oxPC_CD36_ protrude from the surface of oxLDL like whiskers and their avid and specific interactions with CD36 scavenger receptors promotes the uptake of oxLDL by macrophages and smooth muscle cells, resulting in the uncontrolled uptake of toxic oxLDL particles and the subsequent formation of foam cells [19]. Phagocytosis of oxLDL by macrophages is an unregulated process due to lack of negative feedback regulation. Consequently, macrophages take up massive quantities of cholesterol and become foam cells. Upon phagocytosis of oxLDL, the store of cholesteryl esters that are abundant in oxLDL are hydrolyzed to free cholesterol (FC) and free fatty acids inside the macrophage [20]. The endoplasmic reticulum (ER) is sensitive to the increased load of FC, which results in significant ER stress [21]. The signaling pathways activated by ER stress cause macrophage foam cells to undergo apoptosis. Apoptosis and secondary necrosis of foam cells lead to development of atherosclerotic plaques, which can result in acute thrombotic complications and death.

Despite abundant evidence to support the oxidative stress hypothesis of atherosclerosis development, antioxidant approaches in human patients have been mostly ineffective at reducing atherosclerosis. Thus, a greater understanding of the temporal and spatial aspects of ROS production in the tunica intima during oxidative stress is needed. Due to the large array of ROS and their limited lifetimes, it is difficult to pinpoint the specific molecular and biochemical pathways that the oxyradicals perturb. However, some key reactions are well known to contribute to atherogenesis. For example, excess levels of O_2_^·−^ that escape detoxication pathways can react with nitric oxide, resulting in the depletion of this important vasoprotective, anti-inflammatory molecule and the formation of the highly reactive oxidant peroxynitrite [14,22,23] (Figure 1). Chain reactions within lipid membranes initiated by oxyradicals results in unrestrained lipid peroxidation reactions, which leads to the generation of electrophilic α,β-unsaturated aldehydes (e.g., 4-hydroxynonenal, which is abbreviated 4-HNE), aldehydes, and nitroalkenes [24]. These reactive lipids are electrophiles that react either reversibly or irreversibly with nucleophiles in proteins and inhibit their normal activities. The resulting “electrophilic stress” caused by lipid peroxidation is implicated in atherogenesis [24,25].

Cells have homeostatic mechanisms to control the concentrations of ROS and electrophiles. For instance, glutathione (GSH) is a cysteine-containing tripeptide that quenches intracellular ROS, forming oxidized GSSG in the process, and detoxifies electrophiles [9]. Furthermore, peroxidases, such as GSH peroxidase and peroxiredoxins, can detoxify both H_2_O_2_ and organic peroxides. In addition, antioxidant concentrations in cells can be increased by consumption of small biomolecules such as *N*-acetyl cysteine, methionine, or polyphenolic compounds, thereby enhancing intracellular redox homeostasis [24]. Although the biochemical pathways for removing ROS and peroxides have been well characterized, chemopreventative strategies to decrease the production of ROS that result from hypertension, hyperlipidemia, diabetes, autoimmune, and inflammatory disease are still under investigation and a matter of debate [26].

**Figure 2:**
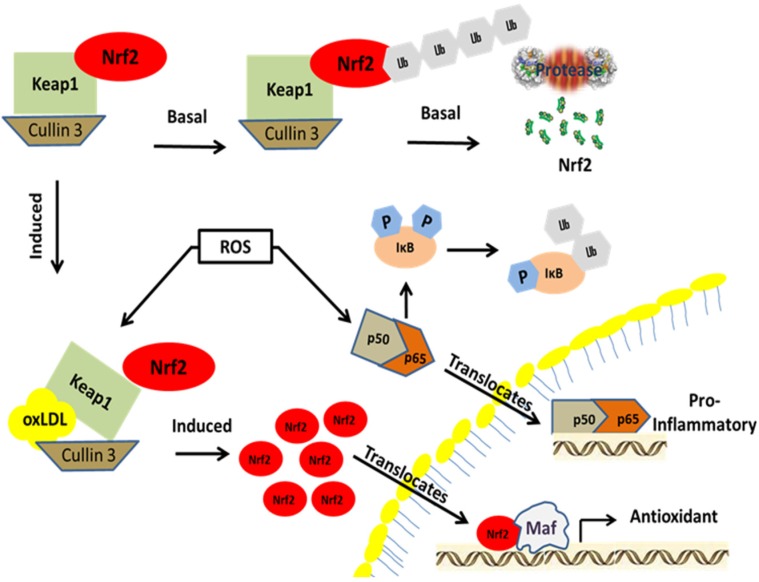
Nrf2 activation pathway. Nrf2 is a member of the bZIP family of transcription factors. It is localized in the cytoplasm under basal conditions where it forms a complex with Keap1 and Cullin 3 until activated. Under basal conditions, Nrf2 is ubiquitinated and degraded by proteosomes. During electrophilic stress induced by either oxLDL or 4-HNE, the cysteine residues in Keap1 are covalently modified leading to the subsequent ubiquitination of Keap1 and its degradation. As Nrf2 builds up in the cytoplasm, it starts to translocate into the nucleus forming a heterodimer with a Maf protein. The transcription factor complex associates with antioxidant response elements upstream of antioxidant and phase II detoxication genes to initiate transcription. Similarly, under basal conditions NFκB forms a complex with its negative regulator IκB in the cytoplasm. During oxidative and inflammatory stress, IκB is phosphorylated by the kinase IKK and subsequently degraded via the ubiquitin-proteosome pathway. This allows NFκB to translocate into the nucleus, resulting in increased expression of genes that encode pro-inflammatory cytokines.

Monocytes adhere to the endothelial cell layer of vessels via their interaction with vascular cell adhesion molecules (VCAM), which are expressed on the endothelial cell surface during inflammation. This causes the development of a positive feedback loop in which NFκB signaling is activated, leading to further endothelial cell dysfunction [27]. The subsequent diapedesis of monocytes through the endothelial cell layer and exposure to M-CSF causes the monocytes to differentiate into intimal macrophages. The differentiation process can occur via classical activation (M1 phenotype), which requires pro-inflammatory stimulants such as interferon-γ and lipopolysaccharide (LPS), resulting in the induction of TNF-α [28]. On the other hand, differentiation can occur via an alternative pathway (M2 phenotype) when macrophages are exposed to anti-inflammatory cytokines, such as IL-4 or IL-13, associated with tissue repair [29]. Although M1 and M2 are the best studied macrophage phenotypes in atherosclerotic plaques, Kuhn *et al.* [30] characterized a Mox phenotype that develops in response to electrophilic oxidized phospholipids and involves the cellular electrophile sensor Nrf2-Keap1 dyad. The Mox phenotype has aspects of both M1 and M2. The heterogeneity of macrophage phenotypes found in atherosclerotic plaques has a major role in the inflammatory process, due to the specific phenotype of the macrophage (M1, M2, or Mox) that contributes to the lesion size, composition, and stability of the plaque [31]. Nrf2 is a vital cellular defense transcription factor that protects against the cytotoxic effects of oxidative and electrophile stress. It is a member of the cap’n-collar family and is a basic leucine zipper transcription factor. Nrf2, under basal conditions, is negatively regulated in the cytoplasm by the cysteine-rich Kelch-like ECH associated protein 1 (Keap1). However, when cysteine residues in Keap1 are covalently modified by electrophiles (such as lipid electrophiles) or oxidants (such as H_2_O_2_), the Keap1-Nrf2 complex dissociates and Nrf2 translocates into the nucleus where it binds to antioxidant response elements (AREs) in the 5′-regulatory region of target genes, thereby initiating transcription (Figure 2). oxPC_CD36_ and 4-HNE each contain an α,β-unsaturated aldehyde moiety, which is formed during lipid peroxidation reactions. 4-HNE is a freely diffusible molecule, whereas oxPC_CD36_ is associated with oxLDL particles. Both are electrophilic molecules that covalently modify cysteines on Keap1, thereby stimulating Nrf2 transactivation activity [24]. Although several studies have described a role for PPAR-γ in regulating CD36 gene expression [13,31,32], it has also been shown that CD36 can be induced by oxPC_CD36_ and 4-HNE via Nrf2 activation [33]. Thus, Nrf2-mediated regulation of CD36 expression in macrophages is a pathway distinct from that of PPAR-γ. Targeting the Nrf2 pathway could be an attractive strategy for inhibiting foam cell formation and atherosclerosis.

## 3. Role of Endocannabinoids (eCBs) in Inflammation

In this section we briefly discuss the role of the eCBs in pathophysiological inflammation; however, for more detailed treatises of this subject, the reader is directed to several excellent publications that describe the eCB system in depth [10,34,35,36,37,38]. ECBs are arachidonoyl-containing lipids that are produced on demand by the metabolism of glycerophospholipid precursors in cell membranes. The best characterized eCBs are 2-arachdonoylglycerol (2-AG) and anandamide (AEA), which are ligands for two G-protein coupled receptors termed cannabinoid (CB) receptors 1 and 2. These eCBs each have similar affinity for the CB_1_ and CB_2_ receptors. The eCBs are local-acting lipid hormones that act in an autocrine or paracrine manner. 2-AG and AEA can be rapidly degraded by the hydrolytic enzymes monoacylglycerol lipase (MAGL) and fatty acid amide hydrolase (FAAH), respectively [34]. The role of the CB_1_ receptor in neurotransmission has been widely studied in the central nervous system. The CB_1_ receptor was identified in the late 1980s by isolating receptors in the brain [39]. More recently these receptors have also been established as having a role in the immune response and may contribute to the dysregulation caused by high blood pressure and inflammation [34]. The CB_1_ receptor is implicated in pro-oxidative stress/pro-inflammatory responses that are associated with CVD [22,40]. The CB_2_ receptor, on the other hand, was cloned in the early 1990s from rat spleen and was also detected in the hematopoietic immune system [41]. On the face of it, the CB_1_ and CB_2_ receptor have similar biochemical functions; they are membrane-bound receptors coupled to a G_i/o_-protein that, when activated, can inhibit adenylyl cyclase and modulate calcium channel activity [35]. However, in contrast to CB_1_, the CB_2_ receptor activation appears to block and modulate several pro-inflammatory effects, suggesting that downstream signaling pathways evoked by the two receptors are divergent.

Macrophages exhibit a profound upregulation in the levels of CB_1_ receptor when exposed to pro-inflammatory and pro-atherogenic stimuli [42], which is implicated in pro-oxidative stress/pro-inflammatory responses associated with CVD [10,33,34,35,36,37]. There is emerging evidence that chronic low doses of Δ^9^-tetrahydrocannabinol (THC), the active component of marijuana, can potentially inhibit the progression of atherosclerotic lesions through interactions involving interferon gamma and decreased macrophage infiltration into the intimal space [43,44]. However, there is conflicting data for THC reported by Chen et al [45], which showed that THC could induce the levels of interferon gamma in the C57BL/6 mouse model, but neither the CB_1_ nor CB_2_ receptor were implicated in this effect. There is evidence suggesting that enhanced 2-AG biosynthesis and subsequent activation of the CB_2_ receptor can reduce the severity of atherosclerotic lesions. Several studies have demonstrated that ligand-mediated activation of the CB_2_ receptor counters inflammation [22]. CB_2_-evoked signaling can downregulate pro-inflammatory cytokines, including tumor necrosis factor-alpha (TNF-α), IL-1β, and monocyte chemoattractant protein-1 (MCP-1), while upregulating the expression of the pattern recognition receptor CR3 (CD18/CD11b) [34]. Furthermore, CB_2_ signaling potentially attenuates the following physiological and molecular effects: inflammatory cell migration, endotoxin-induced oxidative stress, p38-MAPK activation, and TNF-α secretion [40,41]. Following an eight-week high-cholesterol diet feeding regimen, the basal vascular O_2_^·−^ levels were higher and the infiltration of macrophages into vessel wall lesions were more pronounced in the ApoE^−/−^CB_2_^−/−^ double knockout mice compared with ApoE^−/−^ mice [46]. Further, treatment of the ApoE^−/−^ mice with a CB_2_ agonist (JWH 133) reduced the size of atherosclerotic lesions [46]. It was also shown that reconstitution of irradiated ApoE^−/−^ mice with bone marrow derived from ApoE^−/−^CB_2_^−/−^ mice resulted in increased macrophage infiltration into atherosclerotic plaques as compared with irradiated ApoE^−/−^ mice reconstituted with bone marrow from ApoE^−/−^ mice [46]. This last finding indicated that signaling through leukocyte CB_2_ receptors can influence the extent of intimal infiltration of immune cells. In line with these findings, the non-selective CB receptor agonist WIN 55212-2 was shown to reduce atherosclerosis in mice, in part, by reducing the expression of VCAM-1, ICAM-1, and P-selectin in vascular cells [47]. In addition, oxLDL-induced ROS in cultured murine macrophages could be attenuated by WIN 55212-2 [48]. Importantly, the anti-inflammatory and anti-oxidative effects of WIN 55212-2 were completely blocked by the CB_2_ antagonist AM630, implicating the CB_2_ receptor as being responsible for these effects.

Nox activity was found to be attenuated by a CB_2_ agonist in human coronary artery endothelial cells [35]. Mechanistically, this is caused by ligand activation of the CB_2_ receptor, which liberates G_i/o_ to inhibit the activity of the Ras-proximate-1-GTPase activating protein (Rap1-GAP), enabling the small G-protein Rap1 to remain in its GTP-bound “on” state thus causing the disassembly of the Nox holoenzyme complex [42]. CB_2_ activation has also been shown to enhance HDL levels, thereby promoting reverse cholesterol transport (RCT) [49]. RCT is an important pathway by which non-esterified cholesterol is effluxed from peripheral macrophages onto extracellular ApoA1 and mature HDL acceptors for subsequent transport to the liver. Importantly, the de novo generation of HDL particles via RCT helps to increase circulating HDL levels and reduce atheroma development. ECBs are also vital in modulating immune cell proliferation and apoptosis, cytokine production, macrophage-mediated phagocytosis, and inflammatory cell migration during tissue injury [50]. Finally, the eCB ligand 2-AG has been specifically implicated in the modulation of inducible nitric oxide synthase (iNOS), nitric oxide (NO), and ROS production in immune cells [51].

Pharmaceutical targeting of the eCB system shows some promise. For instance, the atherosclerotic lesion size decreased in the abdominal aorta of ApoE^−/−^ mice treated with rimonabant, a CB_1_-specific antagonist [49]. Pharmacological blockade of the CB_1_ receptor or its genetic deletion also enhanced macrophage RCT via ABCA1- and ABCG1-mediated cholesterol transport and reduced atherosclerosis [34,49,52,53]. Rajesh *et al.* [40] demonstrated that chemical blockade of the CB_1_ receptor could attenuate Nox expression, MAPK activation, apoptosis, inflammation, ROS, and fibrosis. In addition, it was shown that CB_1_ receptor inhibition reduced the degree of oxidative stress and decreased the expression of the angiotensin 1 gene in vascular smooth muscle cells [54]. Whereas CB_1_ receptor activation stimulates production of pro-inflammatory mediators, activation of CB_2_ receptors in macrophages attenuated TNF-α production and evoked other anti-inflammatory responses. Several studies indicated that selective activation of CB_2_ may render cardioprotective effects [53,55]. For example, JWH-133, a CB_2_ agonist, reduced O_2_^·−^ generation, increased ERK 1/2 and STAT3 phosphorylation, inhibited chemotaxis, and increased the expression of CR3 on inflammatory cells [34]. On the other hand, when ApoE^−/−^ mice on a high-cholesterol diet were simultaneously treated with the FAAH inhibitor URB597, the level of matrix metaloproteinase-9 increased in the lesion, the vascular collagen content decreased, and plaque vulnerability increased as compared with control vehicle-treated ApoE^−/−^ mice on the same diet [56]. This result suggested that an increased eCB concentration (in this case AEA) in the vessel wall might have negative effects on disease progression. A recent report, however, demonstrated that monoacylglycerol lipase (MAGL) deficiency in ApoE^−/−^ mice on a western-type diet had systemically elevated 2-AG concentrations and exhibited increased atherosclerotic plaque formation mice, but improved plaque stability, compared to control ApoE^−/−^ mice [57].

The eCB system is activated in macrophage-derived foam cells relative to basal macrophages [58]. Several genes that encode components of the eCB system are upregulated. In addition, the concentrations of the ligands for the CB receptor, AEA and 2-AG, are also increased. Selective activation of CB_2_ receptors reduced CD36-dependent oxLDL accumulation and modulated the production of inflammatory cytokines [58].

Therefore, in addition to endothelial dysfunction, hypertension, hyperlipidemia, inflammation, and ROS, the eCB system also appears to be an important factor in atherogenesis [55]. Although the mechanistic role of the eCB system in disease development is still enigmatic, links have been postulated between the eCB system, Nox, oxidative stress, and atherosclerosis [35,59]. Because of the large number of oxyradical species that are produced in cells during vascular injury, which significantly complicates the interpretation of experimental data, it is not a trivial task to identify the pathophysiological pathways induced or enhanced by oxidative stress. Nevertheless, several homeostatic mechanisms appear to have evolved to protect against reactive small molecules, such as ROS and 4-HNE. For example, the enhanced biosynthesis of 2-AG by cells that are “stressed” might be part of a compensatory protective mechanism, via activation of the CB_2_ receptor by 2-AG and downstream signaling, that provides anti-atherogenic effects.

## 4. Diacylglycerol Lipase and Nox

**Figure 3:**
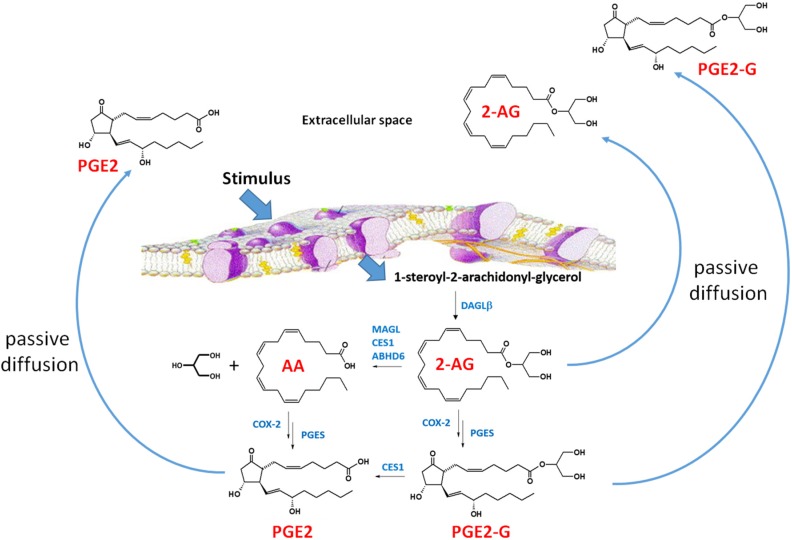
2-Arachidonylglycerol biosynthesis and degradation in macrophages [69]. Following a stimulus, phospholipids are metabolized into 1-steroyl-2-arachidonoyl-glycerol (*sn*-1,2-DAG) by phospholipase C-beta (PLC-β). The lipophilic *sn*-1,2-DAG is localized within the lipid membrane and is subsequently metabolized into 2-AG by the enzyme DAGLβ. 2-AG can be degraded by either hydrolytic enzymes (e.g., monoacylglycerol lipase, carboxylesterase 1 (CES1), and (ABHD6) or by oxygenation cyclooxygenase-2 (COX-2)). COX-2 converts arachidonic acid (AA) to PGG2, which is further metabolized to PGH2 (not shown). PGH2 is subsequently converted to prostaglandins, such as PGE2, by specific synthases (shown is prostaglandin E synthase, PGES). Lipoxygenase (LOX) can also metabolize AA to either 12-hydroxyperoxyeicosa-5,8,10,14-tetraenoic acid (12-HPETE) or 12-hydroxyperoxyeicosa-5,8,10,14-tetraenoic acid (15-HPETE) (not shown). 2-AG, PGE2-G, and PGE2 passively diffuse from the cell into the extracellular space where the lipid mediators act in a paracrine or autocrine manner. The lipid mediators have a short half-time due to their rapid degradation [69]. Figure adapted from [70].

Diacylglycerol (DAG) is a lipid and a second messenger molecule produced by the enzyme phospholipase C-β (PLC-β) [60,61]. DAG is hydrolyzed by *sn*-1-specific diacylglycerol lipases (DAGL) yielding the eCB 2-AG (Figure 3) in response to extracellular stimuli, including angiotensin II, thromboxane A2, platelet activating factor, bradykinin, serotonin, glutamate, and acetylcholine [62,63]. Two isoforms of DAGL (DAGLα and DAGLβ) were identified in humans, on the basis of the conservation of the intron-exon boundary in human DAGL isoforms with those in *Drosophila*, fish, and rodents [64]. DAGLα is found in high concentrations in brain, whereas DAGLβ is found in high concentrations in immune cells and macrophages [65]. Gao *et al.* [66] used DAGLβ^−/−^ knockout mice to show that DAGLβ is a vital enzyme in the biosynthesis of 2-AG. DAGLβ has four transmembrane (4TM) domains, a short cytoplasmic N-terminal sequence, a canonical α/β hydrolase domain that harbors the catalytic residues, and several unconserved loops with potential sites for glycosylation [64]. DAGLβ can also be palmitoylated on a cysteine residue in a regulatory loop [64]. Reversible fatty acyl-posttranslational modifications can modulate protein-protein interactions, cellular trafficking, and substrate access.

*sn*-1,2-DAG is also implicated in the regulation of Nox activity [67], thus DAGLα and DAGLβ are likely to be important hubs in this signaling pathway. Protein kinase C (PKC) is recruited to lipid membranes by the presence of DAG and its kinase activity is activated. This leads to the phosphorylation of the Nox regulatory subunit, p47phox. Elevated concentrations of DAG cause a heightened oxidative response in cells and this signal can be terminated by DAG kinase [67]. DAGLβ also appears to indirectly regulate the production of eicosanoids in the brain, liver, and macrophages by increasing the levels of 2-AG in these tissues and cells [65,68]. When 2-AG hydrolysis was blocked by either chemical inhibition of the principal 2-AG hydrolytic enzyme in brain, MAGL, or its genetic deletion, the production of eicosanoids were decreased and neuroinflammation was reduced in a mouse model [68]. This is because the concentration of the cyclooxygenase substrate, arachidonic acid, available to the eicosanoid biosynthetic enzymes (e.g., cyclooxygenases) had been reduced.

## 5. Endocannabinoid (eCB) Metabolism

The duration of the physiological response to eCBs is regulated by the balance of eCB biosynthetic and degradative pathways. AEA and 2-AG are both degraded by hydrolysis, yielding arachidonic acid as a common metabolite and ethanolamine and glycerol, respectively. In the mouse brain, ~85% of 2-AG hydrolytic activity is due to monoacylglycerol lipase (MAGL), with the remaining balance ascribed to two hydrolytic enzymes, α/β-hydrolase domain (ABHD)-6 and ABHD-12 [71,72]. In addition, 2-AG and prostaglandin glyceryl esters (PG-Gs), which are cyclooxygenase-derived oxygenated products of 2-AG, are also degraded by carboxylesterase 1 (CES1) [73,74]. Further adding to the list of hydrolytic enzymes that metabolize eCBs, Wang *et al.* [74] identified palmitoyl protein thioesterase 1 (PPT1) as an enzyme that can hydrolyze 2-AG. PPT1 is a monomeric lysosomal hydrolase known to hydrolyze thioester bonds that attach long-chain fatty acids to cysteine residues [74,75]. AEA is degraded primarily by FAAH and FAAH-deficient mice have elevated amounts of AEA in tissues compared with wildtype mice [76].

## 6. Conclusions

**Figure 4:**
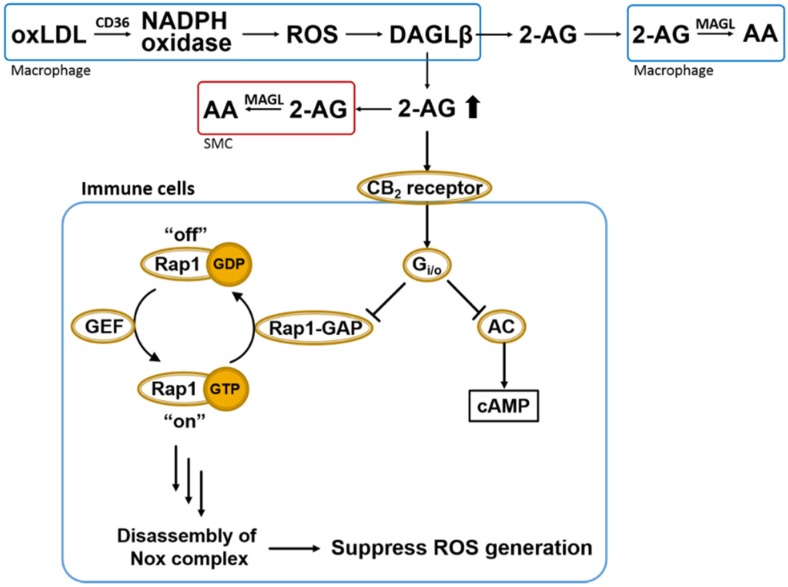
Overall scheme of 2AG biosynthesis/catabolism and anti-oxidative CB_2_-evoked signaling in immune cells. OxLDL-mediated NADPH oxidase (Nox) activation in macrophages leads to ROS-dependent DAGLβ activation and increased biosynthesis of 2AG, which can act in either a paracrine or autocrine manner. Heightened levels of 2AG released in response to increased ROS can stimulate CB_2_ receptors in a compensatory manner to suppress oxidative stress via Gi/o-mediated inactivation of Rap1-GAP (see text for details) [48]. Also shown is the canonical Gi/o-mediated inactivation of adenylate cyclase (AC). Extracellular 2AG is degraded by cells that produce it or by neighboring cells, such as smooth muscle cells (SMCs) and other macrophages. Rap1-GAP, Rap1-GTPase activating protein; GEF, GTP exchange factor; AC, adenylate cyclase; 2-AG, 2-arachidonoylglycerol; AA, arachidonic acid; MAGL, monoacylglycerol lipase; DAGLβ, diacylglycerol lipase β; SMC, smooth muscle cells.

Despite the enormous amount of knowledge that has been gained about atherosclerosis and the primacy of reducing serum cholesterol levels as a treatment modality, cardiovascular disease (CVD) still kills more people in the U.S. than any other disease. Therefore, there is an urgent need to examine as many pathological mechanisms as possible in order to find novel treatment strategies. Occult exposures to environmental toxicants might be adversely impacting numerous CVD patients [77]. Furthermore, complex mixtures of oxidants and electrophiles can be generated in the body following exposures to environmental pollutants and during pathophysiological conditions [78]. These reactive endogenous and exogenous chemicals are crucial etiological factors in CVD, as exemplified by the reactive aldehyde compound acrolein [79]. Macrophages and other cells in the vessel wall respond to this toxic milieu of oxidants and electrophiles by releasing a host of inflammatory mediators and oxyradicals, such as cytokines, chemokines, proteases, eicosanoids, and superoxide anion [80,81]. Furthermore, the quantities of eCBs, such as 2AG, and their cognate receptors are elevated in diseased vessels, indicating that the eCB system is activated in the vessel wall [82], which can elicit negative feedback effects on ROS formation (Figure 4). Thus, how endogenous and exogenous poisons contribute to the initiation and progression of atherosclerosis in vascular cells is a significant research area that will yield novel pharmaceutical strategies for the treatment of this disease.

It is becoming apparent that oxidative stress is both a cause and a consequence of atherosclerosis. O_2_^·−^and H_2_O_2_ are implicated in vascular dysfunction through the initiation of diverse signaling and transcriptional pathways [8,9]. There are arsenals of enzymes in cells that scavenge oxyradicals to reduce the toxic burden of excess ROS. Emerging evidence indicates that the eCBs have the ability to reduce the production of inflammatory mediators in the vascular wall as part of a compensatory response to oxidative stress. Pharmacological inhibition or genetic blockade of the key biosynthetic enzyme DAGLβ reduces the production of the eCB ligand 2-AG, indicating that DAGLβ is a key enzyme in establishing the local eCB concentration, or “tone”, in a tissue or the vascular space. Furthermore, Nox, the “professional ROS generator”, is a promising pharmacological target due to the role it has in O_2_^·−^ production and CVD pathology. Elucidation of the physiological and pathophysiological processes emanating from Nox activation and the role of eCB ligands in attenuating the inflammation and oxidative stress that result from overactive Nox might be a beneficial treatment modality for CVD. Emerging evidence indicates that the increase in local concentration of 2-AG is an adaptive response to oxidative stress because of its antioxidant and anti-inflammatory activities. Therefore, the genetic and lifestyle factors that lead to atherosclerosis development may be counteracted by either increased biosynthesis (via increased DAGLβ activity) or reduced hydrolysis (via decreased MAGL and/or other hydrolytic enzyme activities) of 2-AG.

## References

[1] Ross M., Matthews A., Mangum L. (2014). Chemical atherogenesis: Role of endogenous and exogenous poisons in disease development. Toxics.

[2] Hamilton C.A., Brosnan M.J., McIntyre M., Graham D., Dominiczak A.F. (2001). Superoxide excess in hypertension and aging: A common cause of endothelial dysfunction. Hypertension.

[3] Hansson G.K., Libby P., Tabas I. (2015). Inflammation and plaque vulnerability. J. Intern. Med..

[4] Van der Loo B., Labugger R., Skepper J.N., Bachschmid M., Kilo J., Powell J.M., Palacios-Callender M., Erusalimsky J.D., Quaschning T., Malinski T. (2000). Enhanced peroxynitrite formation is associated with vascular aging. J. Exp. Med..

[5] Brewer T.F., Garcia F.J., Onak C.S., Carroll K.S., Chang C.J. (2015). Chemical approaches to discovery and study of sources and targets of hydrogen peroxide redox signaling through NADPH oxidase proteins. Annu. Rev. Biochem..

[6] Jiang F., Zhang Y., Dusting G.J. (2011). Nadph oxidase-mediated redox signaling: Roles in cellular stress response, stress tolerance, and tissue repair. Pharmacol. Rev..

[7] Al Ghouleh I., Frazziano G., Rodriguez A.I., Csanyi G., Maniar S., St Croix C.M., Kelley E.E., Egana L.A., Song G.J., Bisello A. (2013). Aquaporin 1, Nox1, and Ask1 mediate oxidant-induced smooth muscle cell hypertrophy. Cardiovasc. Res..

[8] Kim Y.W., Byzova T.V. (2014). Oxidative stress in angiogenesis and vascular disease. Blood.

[9] Lambeth J.D., Neish A.S. (2014). Nox enzymes and new thinking on reactive oxygen: A double-edged sword revisited. Annu. Rev. Pathol..

[10] Hopkins P.N. (2013). Molecular biology of atherosclerosis. Physiol. Rev..

[11] Mangum L., Borazjani A., Stokes J., Matthews A.T., Lee J.H., Chambers J., Ross M.K. (2015). Organochlorine insecticides induce NADPH oxidase-dependent reactive oxygen species in human monocytic cells via phospholipase a2/arachidonic acid. Chem. Res. Toxicol..

[12] Park J.-G., Oh G.-T. (2011). The role of peroxidases in the pathogenesis of atherosclerosis. BMB Rep..

[13] Park Y.M., Febbraio M., Silverstein R.L. (2009). CD36 modulates migration of mouse and human macrophages in response to oxidized LDL and may contribute to macrophage trapping in the arterial intima. J. Clin. Investig..

[14] Judkins C.P., Diep H., Broughton B.R., Mast A.E., Hooker E.U., Miller A.A., Selemidis S., Dusting G.J., Sobey C.G., Drummond G.R. (2010). Direct evidence of a role for Nox2 in superoxide production, reduced nitric oxide bioavailability, and early atherosclerotic plaque formation in ApoE^−/−^ mice. Am. J. Physiol. Heart Circ. Physiol..

[15] Drummond G.R., Selemidis S., Griendling K.K., Sobey C.G. (2011). Combating oxidative stress in vascular disease: NADPH oxidases as therapeutic targets. Nat. Rev. Drug Discov..

[16] Tojo T., Ushio-Fukai M., Yamaoka-Tojo M., Ikeda S., Patrushev N., Alexander R.W. (2005). Role of gp91^phox^ (Nox2)-containing NAD(P)H oxidase in angiogenesis in response to hindlimb ischemia. Circulation.

[17] Griendling A.A. (2013). Differential roles of NADPH oxidases in vascular physiology and pathophysiology. Front. Biosci..

[18] Silverstein R.L., Li W., Park Y.M., Rahaman S.O. (2010). Mechanisms of cell signaling by the scavenger receptor CD36: Implications in atherosclerosis and thrombosis. Trans. Am. Clin. Climatol. Assoc..

[19] Salomon R.G., Gu X. (2011). Critical insights into cardiovascular disease from basic research on the oxidation of phospholipids: The gamma-hydroxyalkenal phospholipid hypothesis. Chem. Res. Toxicol..

[20] Ross M.K., Borazjani A., Mangum L.C., Wang R., Crow J.A. (2014). Effects of toxicologically relevant xenobiotics and the lipid-derived electrophile 4-hydroxynonenal on macrophage cholesterol efflux: Silencing carboxylesterase 1 has paradoxical effects on cholesterol uptake and efflux. Chem. Res. Toxicol..

[21] Feng B., Yao P.M., Li Y., Devlin C.M., Zhang D., Harding H.P., Sweeney M., Rong J.X., Kuriakose G., Fisher E.A. (2003). The endoplasmic reticulum is the site of cholesterol-induced cytotoxicity in macrophages. Nat. Cell Biol..

[22] Mukhopadhyay P., Batkai S., Rajesh M., Czifra N., Harvey-White J., Hasko G., Zsengeller Z., Gerard N.P., Liaudet L., Kunos G. (2007). Pharmacological inhibition of CB1 cannabinoid receptor protects against doxorubicin-induced cardiotoxicity. J. Am. Coll. Cardiol..

[23] Heumuller S., Wind S., Barbosa-Sicard E., Schmidt H.H., Busse R., Schroder K., Brandes R.P. (2008). Apocynin is not an inhibitor of vascular NADPH oxidases but an antioxidant. Hypertension.

[24] Levonen A.L., Hill B.G., Kansanen E., Zhang J., Darley-Usmar V.M. (2014). Redox regulation of antioxidants, autophagy, and the response to stress: Implications for electrophile therapeutics. Free Radic. Biol. Med..

[25] Borazjani A., Edelmann M.J., Hardin K.L., Herring K.L., Allen Crow J., Ross M.K. (2011). Catabolism of 4-hydroxy-2-trans-nonenal by THP1 monocytes/macrophages and inactivation of carboxylesterases by this lipid electrophile. Chem. Biol. Interact..

[26] Turrens J.F. (2003). Mitochondrial formation of reactive oxygen species. J. Physiol..

[27] Baker R.G., Hayden M.S., Ghosh S. (2011). Nf-kappaB, inflammation, and metabolic disease. Cell Metab..

[28] Johnson J.L., Newby A.C. (2009). Macrophage heterogeneity in atherosclerotic plaques. Curr. Opin. Lipidol..

[29] Gordon S., Taylor P.R. (2005). Monocyte and macrophage heterogeneity. Nat. Rev. Immunol..

[30] Kuhn A.M., Tzieply N., Schmidt M.V., von Knethen A., Namgaladze D., Yamamoto M., Brune B. (2011). Antioxidant signaling via Nrf2 counteracts lipopolysaccharide-mediated inflammatory responses in foam cell macrophages. Free Radic. Biol. Med..

[31] Adamson S., Leitinger N. (2011). Phenotypic modulation of macrophages in response to plaque lipids. Curr. Opin. Lipidol..

[32] Nagy L., Tontonoz P., Alvarez J.G., Chen H., Evans R.M. (1998). Oxidized LDL regulates macrophage gene expression through ligand activation of ppargamma. Cell.

[33] Ishii T., Itoh K., Ruiz E., Leake D.S., Unoki H., Yamamoto M., Mann G.E. (2004). Role of Nrf2 in the regulation of CD36 and stress protein expression in murine macrophages: Activation by oxidatively modified ldl and 4-hydroxynonenal. Circ. Res..

[34] Pacher P., Steffens S. (2009). The emerging role of the endocannabinoid system in cardiovascular disease. Semin. Immunopathol..

[35] Mackie A.A. (2010). CB2: A cannabinoid receptor with an identity crisis. Br. J. Pharmacol..

[36] Tanasescu R., Constantinescu C.S. (2010). Cannabinoids and the immune system: An overview. Immunobiology.

[37] Booz G.W. (2011). Cannabidiol as an emergent therapeutic strategy for lessening the impact of inflammation on oxidative stress. Free Radic. Biol. Med..

[38] Chiurchiu V., Battistini L., Maccarrone M. (2015). Endocannabinoid signaling in innate and adaptive immunity. Immunology.

[39] Devane W.A., Dysarz F.A., Johnson M.R., Melvin L.S., Howlett A.C. (1988). Determination and characterization of a cannabinoid receptor in rat brain. Mol. Pharmacol..

[40] Rajesh M., Bátkai S., Kechrid M., Mukhopadhyay P., Lee W.S., Horváth B., Holovac E., Cinar R., Liaudet L., Mackie K. (2012). Cannabinoid 1 receptor promotes cardiac dysfunction, oxidative stress, inflammation, and fibrosis in diabetic cardiomyopathy. Diabetes.

[41] Munro S., Thomas K.L., Abu-Shaar M. (1993). Molecular characterization of a peripheral receptor for cannabinoids. Nature.

[42] Han K.H., Lim S., Ryu J., Lee C.W., Kim Y., Kang J.H., Kang S.S., Ahn Y.K., Park C.S., Kim J.J. (2009). CB1 and CB2 cannabinoid receptors differentially regulate the production of reactive oxygen species by macrophages. Cardiovas. Res..

[43] Jiang L.S., Pu J., Han Z.H., Hu L.H., He B. (2009). Role of activated endocannabinoid system in regulation of cellular cholesterol metabolism in macrophages. Cardiovas. Res..

[44] Steffens S., Veillard N.R., Arnaud C., Pelli G., Burger F., Staub C., Karsak M., Zimmer A., Frossard J.L., Mach F. (2005). Low dose oral cannabinoid therapy reduces progression of atherosclerosis in mice. Nature.

[45] Chen W., Crawford R.B., Kaplan B.L., Kaminski N.E. (2015). Modulation of HIVGP120 antigen-specific immune responses in vivo by delta9-tetrahydrocannabinol. J. Neuroimmune Pharmacol..

[46] Hoyer F.F., Steinmetz M., Zimmer S., Becker A., Lutjohann D., Buchalla R., Zimmer A., Nickenig G. (2011). Atheroprotection via cannabinoid receptor-2 is mediated by circulating and vascular cells in vivo. J Mol. Cell. Cardiol..

[47] Zhao Y., Yuan Z., Liu Y., Xue J., Tian Y., Liu W., Zhang W., Shen Y., Xu W., Liang X. (2010). Activation of cannabinoid CB2 receptor ameliorates atherosclerosis associated with suppression of adhesion molecules. J. Cardiovasc. Pharmacol..

[48] Hao M.X., Jiang L.S., Fang N.Y., Pu J., Hu L.H., Shen L.H., Song W., He B. (2010). The cannabinoid WIN55,212–2 protects against oxidized LDL-induced inflammatory response in murine macrophages. J. Lipid Res..

[49] Sugamura K., Sugiyama S., Fujiwara Y., Matsubara J., Akiyama E., Maeda H., Ohba K., Matsuzawa Y., Konishi M., Nozaki T. (2010). Cannabinoid 1 receptor blockade reduces atherosclerosis with enhances reverse cholesterol transport. J. Atheroscler. Thromb..

[50] Chouinard F., Lefebvre J.S., Navarro P., Bouchard L., Ferland C., Lalancette-Hebert M., Marsolais D., Laviolette M., Flamand N. (2011). The endocannabinoid 2-arachidonoyl-glycerol activates human neutrophils: Critical role of its hydrolysis and de novo leukotriene B_4_ biosynthesis. J. Immunol..

[51] Pacher P., Beckman J.S., Liaudet L. (2007). Nitric oxide and peroxynitrite in health and disease. Physiol. Rev..

[52] Tsubakio-Yamamoto K., Matsuura F., Koseki M., Oku H., Sandoval J.C., Inagaki M., Nakatani K., Nakaoka H., Kawase R., Yuasa-Kawase M. (2008). Adiponectin prevents atherosclerosis by increasing cholesterol efflux from macrophages. Biochem. Biophys. Res. Commun..

[53] Pacher P., Kunos G. (2013). Modulating the endocannabinoid system in human health and disease—successes and failures. FEBS J..

[54] Tiyerili V., Zimmer S., Jung S., Wassmann K., Naehle C.P., Lutjohann D., Zimmer A., Nickenig G., Wassmann S. (2010). CB1 receptor inhibition leads to decreased vascular AT1 receptor expression, inhibition of oxidative stress and improved endothelial function. Basic Res. Cardiol..

[55] Pacher P., Mechoulam R. (2011). Is lipid signaling through cannabinoid 2 receptors part of a protective system?. Prog. Lipid Res..

[56] Hoyer F.F., Khoury M., Slomka H., Kebschull M., Lerner R., Lutz B., Schott H., Lutjohann D., Wojtalla A., Becker A. (2014). Inhibition of endocannabinoid-degrading enzyme fatty acid amide hydrolase increases atherosclerotic plaque vulnerability in mice. J. Mol. Cell. Cardiol..

[57] Vujic N., Schlager S., Eichmann T.O., Madreiter-Sokolowski C.T., Goeritzer M., Rainer S., Schauer S., Rosenberger A., Woelfler A., Doddapattar P. (2016). Monoglyceride lipase deficiency modulates endocannabinoid signaling and improves plaque stability in ApoE-knockout mice. Atherosclerosis.

[58] Chiurchiu V., Lanuti M., Catanzaro G., Fezza F., Rapino C., Maccarrone M. (2014). Detailed characterization of the endocannabinoid system in human macrophages and foam cells, and anti-inflammatory role of type-2 cannabinoid receptor. Atherosclerosis.

[59] Anilkumar N., Weber R., Zhang M., Brewer A., Shah A.M. (2008). Nox4 and nox2 NADPH oxidases mediate distinct cellular redox signaling responses to agonist stimulation. Arterioscler. Thromb. Vasc. Biol..

[60] Shonesy B.C., Winder D.G., Patel S., Colbran R.J. (2015). The initiation of synaptic 2-AG mobilization requires both an increased supply of diacylglycerol precursor and increased postsynaptic calcium. Neuropharmacology.

[61] Signorello M.G., Giacobbe E., Segantin A., Avigliano L., Sinigaglia F., Maccarrone M., Leoncini G. (2011). Activation of human platelets by 2-arachidonoylglycerol: Role of PKC in NO/cGMP pathway modulation. Curr. Neurovasc. Res..

[62] Rouzer C.A., Ghebreselasie K., Marnett L.J. (2002). Chemical stability of 2-arachidonylglycerol under biological conditions. Chem. Phys. Lipids.

[63] Astarita G., Piomelli D. (2009). Lipidomic analysis of endocannabinoid metabolism in biological samples. J. Chromatogr. B.

[64] Reisenberg M., Singh P.K., Williams G., Doherty P. (2012). The diacylglycerol lipases: Structure, regulation and roles in and beyond endocannabinoid signalling. Philos. Trans. B.

[65] Hsu K.L., Tsuboi K., Adibekian A., Pugh H., Masuda K., Cravatt B.F. (2012). DAGLβ inhibition perturbs a lipid network involved in macrophage inflammatory responses. Nat. Chem. Biol..

[66] Gao Y., Vasilyev D.V., Goncalves M.B., Howell F.V., Hobbs C., Reisenberg M., Shen R., Zhang M.Y., Strassle B.W., Lu P. (2010). Loss of retrograde endocannabinoid signaling and reduced adult neurogenesis in diacylglycerol lipase knock-out mice. J. Neurosci..

[67] Schlam D., Bohdanowicz M., Chatgilialoglu A., Steinberg B.E., Ueyama T., Du G., Grinstein S., Fairn G.D. (2013). Diacylglycerol kinases terminate diacylglycerol signaling during the respiratory burst leading to heterogeneous phagosomal NADPH oxidase activation. J. Biol. Chem..

[68] Nomura D.K., Morrison B.E., Blankman J.L., Long J.Z., Kinsey S.G., Marcondes M.C., Ward A.M., Hahn Y.K., Lichtman A.H., Conti B. (2011). Endocannabinoid hydrolysis generates brain prostaglandins that promote neuroinflammation. Science.

[69] Rouzer C.A., Marnett L.J. (2011). Endocannabinoid oxygenation by cyclooxygenases, lipoxygenases, and cytochromes P450: Cross-talk between the eicosanoid and endocannabinoid signaling pathways. Chem. Rev..

[70] Rouzer C.A., Marnett L.J. (2008). Non-redundant functions of cyclooxygenases: Oxygenation of endocannabinoids. J. Biol. Chem..

[71] Blankman J.L., Simon G.M., Cravatt B.F. (2007). A comprehensive profile of brain enzymes that hydrolyze the endocannabinoid 2-arachidonoylglycerol. Chem. Biol..

[72] Carr R.L., Graves C.A., Mangum L.C., Nail C.A., Ross M.K. (2013). Low level chlorpyrifos exposure increases anandamide accumulation in juvenile rat brain in the absence of brain cholinesterase inhibition. Neurotoxicology.

[73] Xie S., Borazjani A., Hatfield M.J., Edwards C.C., Potter P.M., Ross M.K. (2010). Inactivation of lipid glyceryl ester metabolism in human THP1 monocytes/macrophages by activated organophosphorus insecticides: Role of carboxylesterases 1 and 2. Chem. Res. Toxicol..

[74] Wang R., Borazjani A., Matthews A.T., Mangum L.C., Edelmann M.J., Ross M.K. (2013). Identification of palmitoyl protein thioesterase 1 in human THP1 monocytes and macrophages and characterization of unique biochemical activities for this enzyme. Biochemistry.

[75] Lu J.Y., Hofmann S.L. (2006). Thematic review series: Lipid posttranslational modifications. Lysosomal metabolism of lipid-modified proteins. J. Lipid Res..

[76] Cravatt B.F., Demarest K., Patricelli M.P., Bracey M.H., Giang D.K., Martin B.R., Lichtman A.H. (2001). Supersensitivity to anandamide and enhanced endogenous cannabinoid signaling in mice lacking fatty acid amide hydrolase. Proc. Natl. Acad. Sci. U S A..

[77] Ramos K.S., Partridge C.R., Teneng I. (2007). Genetic and molecular mechanisms of chemical atherogenesis. Mutat. Res..

[78] Liebler D.C. (2006). The poisons within: Application of toxicity mechanisms to fundamental disease processes. Chem. Res. Toxicol..

[79] Srivastava S., Sithu S.D., Vladykovskaya E., Haberzettl P., Hoetker D.J., Siddiqui M.A., Conklin D.J., D'Souza S.E., Bhatnagar A. (2011). Oral exposure to acrolein exacerbates atherosclerosis in apoE-null mice. Atherosclerosis.

[80] Laskin D.L., Sunil V.R., Gardner C.R., Laskin J.D. (2011). Macrophages and tissue injury: Agents of defense or destruction?. Annu. Rev. Pharmacol. Toxicol..

[81] Soehnlein O., Weber C. (2009). Myeloid cells in atherosclerosis: Initiators and decision shapers. Semin. Immunopathol..

[82] Sugamura K., Sugiyama S., Nozaki T., Matsuzawa Y., Izumiya Y., Miyata K., Nakayama M., Kaikita K., Obata T., Takeya M. (2009). Activated endocannabinoid system in coronary artery disease and antiinflammatory effects of cannabinoid 1 receptor blockade on macrophages. Circulation.

